# Menopause and Risk-Taking Behaviours: A Cross-Sectional, Online Survey

**DOI:** 10.1192/bjo.2024.232

**Published:** 2024-08-01

**Authors:** Dan Reisel, Aini Kamal, Sarah Glynne, Louise Newson

**Affiliations:** Newson Health, Stratford-upon-Avon, United Kingdom

## Abstract

**Aims:**

Limited data suggest that negative mood symptoms in the menopause transition may be associated with a higher prevalence of alcohol misuse and other risk-taking behaviours in menopausal women. Excessive alcohol consumption can exacerbate menopausal symptoms, reduce quality of life and is associated with chronic morbidity that overlaps with the consequences of long-term oestrogen deficiency (such as osteoporosis and cardiovascular disease). The aim of this survey was to explore the impact of mental ill-health on alcohol consumption and gambling habits in menopausal women.

**Methods:**

We constructed an anonymous survey consisting of multiple-choice and free-text questions. The survey was distributed online via social media channels on the 22 August 2023 and was open for 6 weeks. All perimenopausal and menopausal women were invited to participate. Responses were collected using the Qualtrics survey platform and analysed in Excel for descriptive statistics.

**Results:**

1,178 responses were submitted. One in three women reported drinking more alcohol during the perimenopause/menopause; 15% of women drink more than the recommended maximum of 14 units per week, and 24% (286) are spending up to £50 per week on alcohol. 70% (332) cited anxiety, stress, and/or depression as the reason for their increased alcohol consumption, whilst 29% (135) said they drank to alleviate menopause symptoms. Further, 5% (54) of respondents admitted gambling more since the onset of perimenopause/menopause; 43% (27) said it was due to anxiety, stress, and/or depression, whilst 13% (9) said they do so to help manage their menopause symptoms.

**Conclusion:**

This anonymous, cross-sectional survey found evidence of an association between menopause and addiction. Increased awareness of this association should facilitate earlier recognition and more timely access to support and effective treatment for addiction, including hormone replacement therapy to treat menopausal symptoms that may underlie and/or exacerbate unhealthy lifestyle behaviours.

